# Supplementary material for: Whole Cell Luminescence-Based Screen for Inhibitors of the Bacterial Sec Machinery
Source: Biochemistry. 2024 Aug 29;63(18):2344–51. doi: 10.1021/acs.biochem.4c00264 (PMC11411707; doi:10.1021/acs.biochem.4c00264)
Supplement: Supplementary file 1 — bi4c00264_si_001.pdf [file bi4c00264_si_001.pdf]

A whole cell luminescence-based screen for inhibitors of the bacterial Sec machinery

Tia Salter, Ian Collinson & William J. Allen**

Address: School of Biochemistry, University of Bristol, University Walk, Bristol BS8 1TD, United Kingdom

Supporting Information

Figure S1

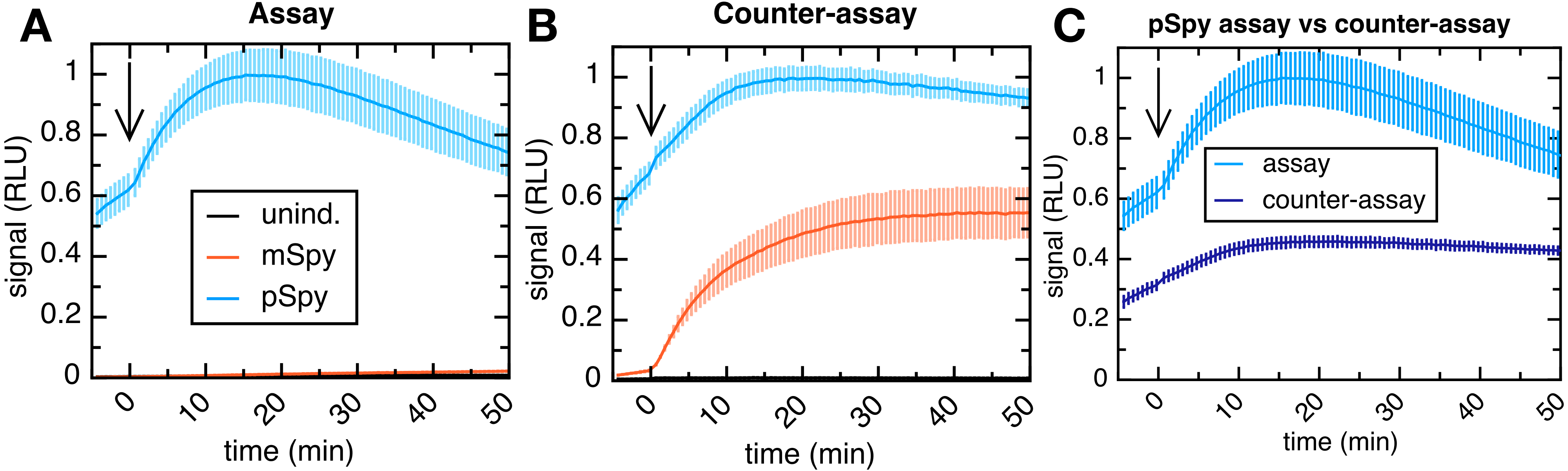

Figure S1. Time courses for (**A**) assay and (**B**) counter assay, normalised to pSpy. Uninduced cells are shown in black, mSpy in red and pSpy in blue. Addition of EDTA and lysozyme (time = 0) is indicated by an arrow. (**C**) Assay and counter-assay for pSpy, normalised to maximum assay signal.

The data in Fig. 1D-E are the maximum values taken from panels A-B.

Figure S2

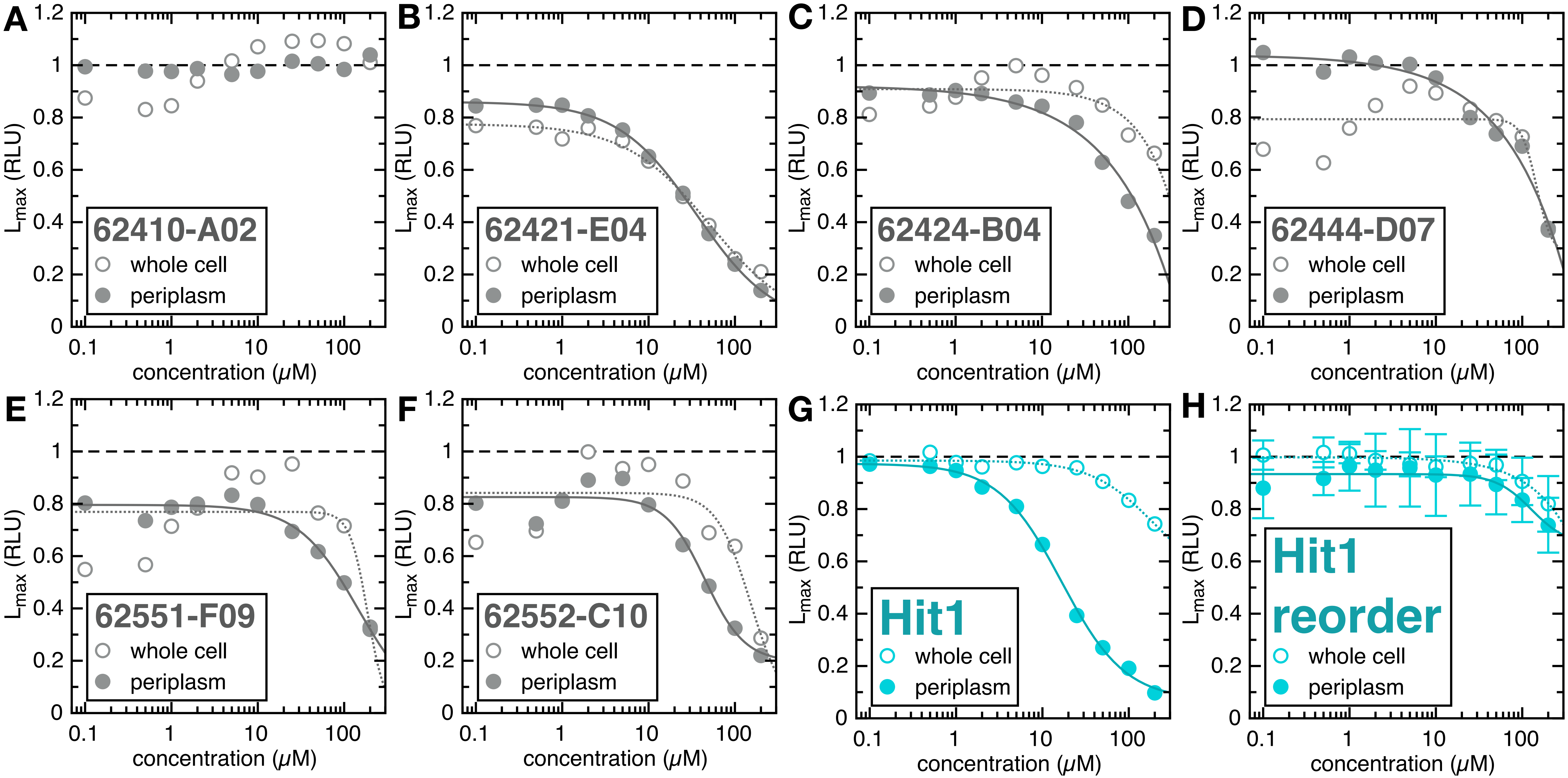

Figure S2. Dose response curves for non-inhibitors of secretion. **(A)** was a false positive from the screen. **(B-F)** were non-specific inhibitors, and **G** was initially identified as an inhibitor (Hit1), but upon repurchase no longer inhibited secretion at all **(H)**.
